# Growth Propagation of Liquid Spawn on Non-Woven Hemp Mats to Inform Digital Biofabrication of Mycelium-Based Composites

**DOI:** 10.3390/biomimetics10010033

**Published:** 2025-01-08

**Authors:** Andreas Biront, Mart Sillen, Patrick Van Dijck, Jan Wurm

**Affiliations:** 1 Research Group Architectural Engineering, Department of Architecture, KU Leuven, 3001 Leuven, Belgium; andreas.biront@kuleuven.be; 2 Laboratory of Molecular Cell Biology, Institute of Botany and Microbiology, KU Leuven, Kasteelpark Arenberg 31, 3001 Leuven, Belgium; mart.sillen@kuleuven.be

**Keywords:** mycelium-based composites, non-woven mats, hemp substrate, digital biofabrication, growth characteristics, manufacturing variables, liquid spawn, *Ganoderma lucidum*, *Pleurotus ostreatus*

## Abstract

Mycelium-based composites (MBCs) are highly valued for their ability to transform low-value organic materials into sustainable building materials, offering significant potential for decarbonizing the construction sector. The properties of MBCs are influenced by factors such as the mycelium species, substrate materials, fabrication growth parameters, and post-processing. Traditional fabrication methods involve combining grain spawn with loose substrates in a mold to achieve specific single functional properties, such as strength, acoustic absorption, or thermal insulation. However, recent advancements have focused on digital biofabrication to optimize MBC properties and expand their application scope. Despite these developments, existing research predominantly explores the use of grain spawn inoculation, with little focus on liquid spawn. Liquid spawn, however, holds significant potential, particularly in digital biofabrication, due to its ease of deposition and greater precision compared with grains. This paper, part of a digital biofabrication framework, investigates the growth kinetics of *Ganoderma lucidum* and *Pleurotus ostreatus* on hemp non-woven mats, offering flexibility and mold-free fabrication using liquid inoculation. By integrating mycelium growth kinetics into digital biofabricated materials, researchers can develop more sustainable, efficient, and specialized solutions using fewer resources, enhancing the adaptability and functionality of MBCs. The experiment involved pre-cultivating *P. ostreatus* and *G. lucidum* in yeast peptone dextrose (YPD) and complete yeast media (CYM) under static (ST) and shaking (SH) conditions. Four dilutions (1:10, 1:2, 1:1, and 2:1) were prepared and analyzed through imagery to assess growth kinetics. Results showed that lower dilutions promoted faster growth with full coverage, while higher dilutions offered slower growth with partial coverage. SH conditions resulted in slightly higher coverage and faster growth. To optimize the control of material properties within the digital biofabrication system, it is recommended to use CYM ST for *P. ostreatus* and YPD SH for *G. lucidum*, as their growth curves show clear separation between dilutions, reflecting distinct growth efficiencies and speeds that can be selected for desired outcomes.

## 1. Introduction

In recent years, research has put more focus on investigating the fabrication of mycelium-based composites (MBCs) for building interior applications, as they hold the potential to be more affordable compared with petroleum-based materials [1,2,3,4,5]. These composites are biofabricated through the growth of fungal mycelium, forming a dense network of hyphae that binds organic substrate materials into new composites. The performance of these materials is dependent on four key factors: the mycelium species, substrate material, fabrication growth parameters, and post-processing of the material [1,6]. The usage of MBCs in construction is mainly focused on their use for thermal insulation and as acoustic absorbers due to their porous structures, yet researchers are investigating to improve their mechanical properties such that they can be used as load-bearing components. MBC properties can be engineered by altering the factors to improve the performance and application range [7,8]. To broaden the application field of MBC understanding, these four key factors will enable researchers to develop more standardized materials for the construction sector. To do this, we need to understand how the current biofabrication process works and build further on this knowledge to scale up fabrication processes.

Alaneme, K.K. et al. [6] summarized the traditional and most common biofabrication process by selecting the fungal specie and pre-cultivating the mycelium on a grain-based substrate, which serves to enhance mycelial biomass production. This preculture is then combined with sterilized loose substrate materials, selected based on the desired functionality of the final product, in a mold. The mixture is incubated under species-specific conditions, including temperature and relative humidity, to optimize mycelial growth. During incubation, the mycelium secretes enzymes (e.g., cellulases, hemicellulases, and ligninases) that degrade the lignocellulosic components of the organic material [9]. This enzymatic breakdown releases nutrients, allowing the mycelium to grow further and form a dense interconnected network throughout the substrate. After a defined incubation period, the mycelium-based component is removed from the incubator and typically oven-dried. This step serves to deactivate the mycelium, halting further growth, and stabilizing the material for its intended application.

To scale up and further improve the understanding of material properties, identifying the most effective inoculation method is a critical first step. Grain spawn as an inoculation technique has been widely studied and mapped in terms of fabrication processes compared with liquid spawn. However, liquid spawn has been shown to improve mechanical strength over grain spawn. Holt et al. and Yang et al. [10,11] demonstrated that liquid inoculation enhances the flexural Young’s modulus and compressive strength. This improvement over grain spawn may result from the easier distribution of mycelium within the substrate’s center [11].

From a digital biofabrication perspective, liquid inoculation offers advantages due to its more workable nature, making it suitable for deposition or injection. It allows for greater precision and control. Therefore, we will investigate liquid spawn as an inoculation technique and its effect on mycelium kinetics. The application possibilities of liquid spawn in mycelium biofabrication expand the potential for digital fabrication techniques such as 3D printing, including direct ink writing and fused deposition modeling. To scale up the production of MBCs, researchers have increasingly focused on digital biofabrication methods to enhance growth, improve material properties, and shape mycelium materials [12,13,14,15,16,17]. Currently, the primary focus of digital fabrication methods is on additive manufacturing (AM).

Over the past decade, AM has been widely adopted to produce three-dimensional products using a gradual layer-by-layer material deposition process. AM is categorized into liquid-based, solid-based, and powder-based techniques [18], with newer subcategories emerging, such as slurry-based 3D printing, which combines elements of multiple AM types [19]. When applied to mycelium-based materials, AM involves creating composites that integrate lignocellulosic substrates with fungal inoculation either during or after the manufacturing process. Most experiments in the literature employ the direct ink writing technique, where an extrudable paste made of lignocellulosic material is used to fabricate scaffolding structures. These studies explore inoculating the paste either before or after the fabrication process [20,21,22,23,24,25]. However, no fabrication techniques have yet investigated the direct deposition of liquid spawn onto or within non-printed substrate material.

We introduce the use of non-woven mats as a promising substrate material, offering several advantages, including greater design flexibility, initial mechanical properties, and cost reduction, as they eliminate the need for full surface molds during production. We hypothesize that liquid spawn can be more accurately applied to non-woven mats, promoting uniform mycelial growth and enabling better control over the growth mechanisms of the mycelium across the substrate. This paper explores the deposition of liquid spawn on non-woven hemp mats. As a starting point for cultivating liquid spawn, we will follow the findings of Appels V. W. et al., who demonstrated that the highest biomass yield occurs in a dark environment with low CO_2_ levels [26]. Our choice of hemp is because of its growing availability in Belgium and neighboring countries, making it a regional resource [27]. The goal of this paper is to develop a protocol to produce liquid spawn for digital biofabrication and understand the growth kinetics of liquid spawn on non-woven mats. Through the understanding of mycelium growth, researchers can harness this feature to design and fabricate with. This is the first study undertaking an analysis of liquid inoculation on non-woven hemp mats by characterizing its growth kinetics.

## 2. Materials and Methods

The methodological approach focused on monitoring the growth of the local liquid inoculation of *G. lucidum* and *P. ostreatus* on non-woven hemp mats. The effect of two liquid nutrient media in a static and shaking incubation on mycelium growth as a preculture and respective dilutions were mapped and compared by analyzing the surface colonization rate. During this approach, the ease of inoculation was compared to understand its usability for digital biofabrication.

This experiment was part of a two-stage protocol designed to cultivate mycelium in liquid media, followed by using these precultures to grow mycelium on non-woven hemp mats. The procedure consisted of two primary stages, carried out in succession:

1. Development of mycelium-based liquid spawn.
2. Monitoring growth on non-woven mats.

### 2.1. Fungal Species and Materials

*G. lucidum* (M9726) and *P. ostreatus* (M2191) are both white-rot species and their grain spawn was purchased from Mycelia bvba (Veldeken 38A, 9850 Nevele, Belgium). The species were conserved on a grain mixture at 4 °C in a breathing Microsac 5 L bag (Sac O2 nv, Nevele, Belgium). The studies were performed on 100% natural non-woven hemp mats “HempFlax Felt” obtained from HempFlax (Groningen, The Netherlands).

### 2.2. Preparation of Liquid Spawn

Grain spawn of both fungal strains were inoculated on a yeast extract peptone dextrose (YPD) 2% glucose agar plate. Subsequently, these plates were incubated at 27 °C for 7 days. Once the mycelium was fully matured on the agar plates, liquid spawn was prepared in either a liquid YPD (2% glucose) or liquid complete yeast medium (CYM).

The agar plates were prepared by mixing 10 g of granulated yeast extract, 20 g of bacteriological peptone, 50 mL of 40% glucose, and 20 g of agar into 950 mL of demineralized water. The liquid YPD medium was made using the same ingredients but without the addition of agar. The liquid CYM medium consisted of 2 g of granulated yeast extract, 2 g of bacteriological peptone, 0.5 g of MgSO_4_, 1 g of K_2_HPO_4_, 0.46 g of KH_2_PO_4_, and 50 mL of 40% glucose, also added to 950 mL of demineralized water. All solutions were sterilized in an autoclave for 15 min at 121 °C, with the glucose added afterward to each solution. The YPD agar was poured into petri dishes and allowed to solidify. All three media were then stored in a refrigerator at 4 °C for the duration of the experiment.

**Figure 1:**
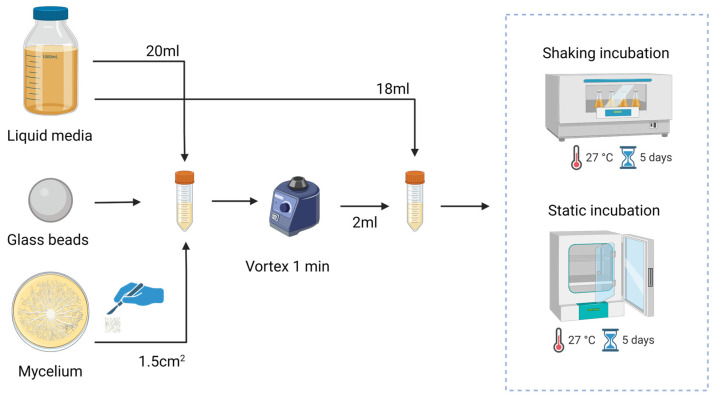
Growth protocol for liquid mycelium spawn by homogenizing mycelium to make a 10 wt % mycelium liquid solution that is statically or shakenly grown for 5 days.

To create the liquid spawn, seen in Figure 1, a mycelium section of 1.5 cm^2^ was transferred from the agar plate to a falcon tube containing glass beads and 20 mL of liquid media. To ensure thorough homogenization of the mycelium, the falcon tube was vortexed. From the homogenized mycelium, 2 mL was transferred to a new falcon tube containing 18 mL of the according medium and placed at 27 °C in a static or shaking incubator for a period of 5 days.

### 2.3. Inoculation of Both Mycelium Species on Non-Woven Hemp Mats

The hemp non-woven mats were cut into 5 cm^2^ squares. After cutting the non-woven mats, the dry weight was measured, enabling the calculation of moisture content after the sterilization (121 °C for 15 min). After 5 days of growing liquid spawn statically (ST) and shakenly (SH), 4 different precultures were made—ST YPD, SH YPD, ST CYM, and SH CYM—for both mycelium strains. After preparing the preculture, 1:10, 1:2, 1:1, and 2:1 dilutions were made. The dilution ratios were chosen to systematically explore the influence of varying mycelium spawn concentrations on the experiment. This approach allowed us to investigate whether the concentration of mycelium spawn had a significant impact on the growth kinetics and to identify potential trends across a range of dilutions. The selected ratios also ensured a clear comparison between larger and smaller dilutions, enabling a comprehensive understanding of the effect of spawn concentration. It was necessary to vortex the SH condition, due to the pellet forming, before making the 4 dilutions. From each dilution, three biological repeats were made for each dilution, and each non-woven mat was inoculated with 3 mL applied to the center, with three technical repeats conducted per mat. After inoculation, all samples were stored in an incubator at 27 °C for 14 days. After 14 days of incubation, the mats were dried at 60 °C for 15 h to terminate the mycelium growth, allowing for their preservation for future research.

### 2.4. Surface Colonization Rate Measurement and Analysis

**Figure A1:**
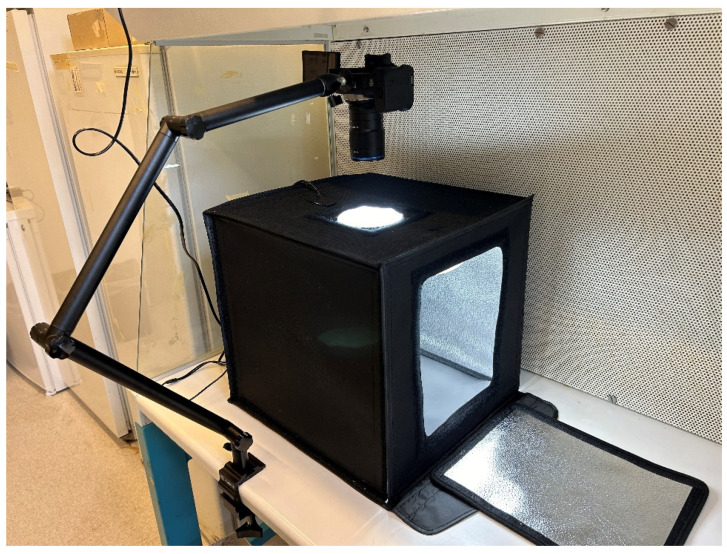
Setup of the photo box in the laminar flow to take pictures of the samples.

To monitor mycelium growth during incubation, the samples were photographed every 24 h for 14 days. A photo box setup was arranged beneath the laminar flow hood, shown in Figure A1 (Appendix A), to minimize the risk of contamination during photography. The images were captured with a Canon EOS R10 and a CA-Dreamer Macro 2× lens, positioned 46 cm above the samples. The aperture was set to f/8 with a focus distance of 0.37 to 1 m. A red mark on the petri dish and tape in the photo box ensured proper alignment of the samples.

Analysis of the mycelium extension rate was conducted by measuring the surface area covered by the mycelium. Images were imported into ImageJ, where the scale was set using the petri dish diameter. The average diameter was calculated by averaging both the horizontal and vertical measurements. From this average diameter, the area was calculated and then expressed as a percentage of the total area of the mat on which it was growing. This percentage was then plotted against time (days) to generate the growth curve.

## 3. Results

To obtain more insights into whether the growth of mycelium strains was dependent on preculture medium, shaking or static conditions, and the dilution of inoculants on non-woven mats, we aimed to identify differences in growth efficiency. This analysis was conducted because understanding the growth of liquid spawn on non-woven mats would pave the way for depositing with and controlling its growth through robotic manipulations during incubation. This was the first step in understanding how this production method could be achieved using mycelium liquid spawn.

### 3.1. Mycelium Growth in Liquid Media

**Figure 2:**
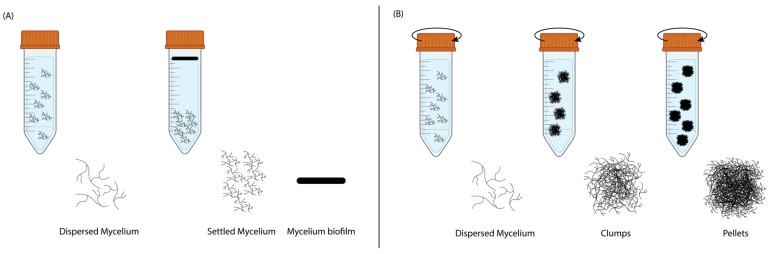
The growth mechanics of mycelium during liquid state fermentation in (**A**) static and (**B**) shaking incubation.

**Figure A2:**
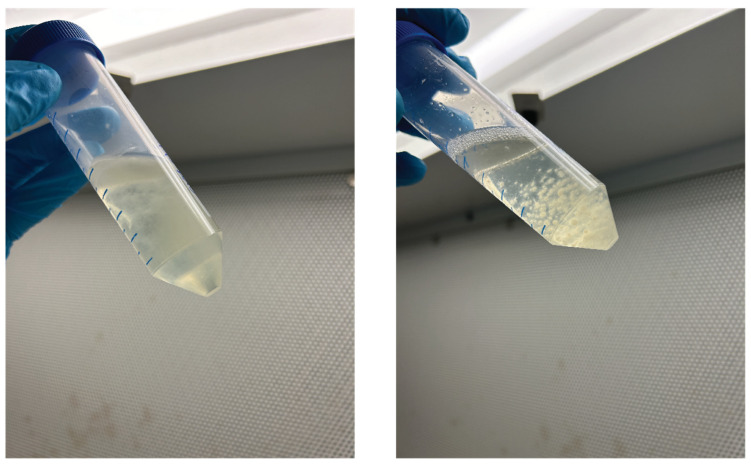
The visual characteristics of mycelium growth in a static (**left**) or shaking (**right**) condition.

Mycelium exhibited distinct growth mechanisms under ST and SH conditions, as shown in Figure 2 and Figure A2 (Appendix A). During ST incubation, the inoculated mycelial hyphae began to branch, interconnect, and form a dispersed mycelial network in the liquid medium. Over time, in the absence of agitation, gravity caused the denser hyphal network to settle at the bottom of the falcon tube, forming a compact settled mycelium layer. Concurrently, a pure mycelium biofilm developed at the liquid–air interface. This biofilm formation was driven by the mycelium’s natural tendency to seek oxygen, which is essential for aerobic growth, with the liquid surface offering the most oxygen-rich environment. As growth continued, the hyphal network at the interface thickened, eventually forming a cohesive and structured biofilm.

In contrast, during SH incubation, shaking introduced turbulence that maintained a homogeneous distribution of nutrients and oxygen throughout the liquid medium. This agitation prevented the formation of a biofilm at the liquid– air interface, as oxygen was dissolved directly into the liquid. Under these conditions, the mycelial hyphae branched and interconnected, forming a dispersed mycelial network. The shaking speed inhibited the mycelium from settling at the bottom and instead promoted the aggregation of hyphae into clumps. Over time, these clumps matured into compact spherical structures known as mycelial pellets.

### 3.2. Mycelium Growth and Surface Colonization

The results show that the dilution and liquid media composition influenced the growth kinetics. To assess the influence of the dilution and liquid media composition, different growing conditions and dilutions were made and compared with each other. The liquid spawn growth curves began at zero due to the absence of observable growth on the day of inoculation. The main results from the growth curves of both species, as expected, showed that the lower the dilution the faster and more likely the hyphae will achieve 100% coverage. The liquid media achieved full coverage between day 7 and day 11. Coverage on day 14 suggested that *G. lucidum* was more reliable than *P. ostreatus* to achieve 100% coverage over all conditions. The growth curves of both species indicated that CYM improved the growth of hyphae across all dilutions compared with YPD, and a closer examination revealed that the shaking conditions achieved faster growth rate and faster full coverage over their static counterpart.

#### 3.2.1. Growth Kinetics of *P. ostreatus* on Non-Woven Hemp Mats

**Figure 3:**
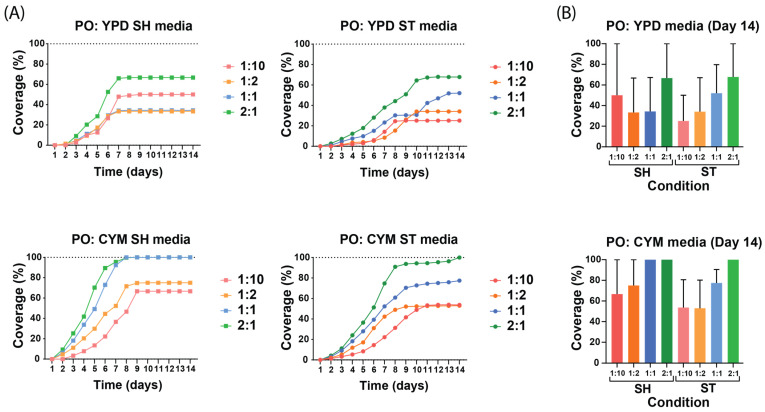
Growth curves of *P. ostreatus* under different preculture conditions and dilutions. All values are presented as mean from n ≥ 2 independent samples and 1:10 to 2:1 indicate the dilution of mycelium in the liquid media. (**A**) Growth curves of liquid spawn of each medium and each condition. The growth curves highlight how these variables affect the growth kinetics across different media and conditions. (**B**) Coverage percentage of all static and shaking conditions on the 14th day of growth of *P. ostreatus* in YPD and CYM media.

When comparing the liquid media and dilutions for *P. ostreatus* (Figure 3), the preculture in the CYM medium was found to enhance growth more effectively than the preculture in YPD, and mycelium from shaking conditions achieved faster growth rate and faster full coverage than the static conditions. The SH media of *P. ostreatus* showed similar growth rates, achieving the stationary phase between day 7 and day 9, whereas the ST media achieved it between day 8 and day 11. Interestingly, when examining the differences in culture media, the cultivation of *P. ostreatus* in YPD media did not support full coverage of the hemp mats, while CYM media achieved full coverage. The comparison between static and shaking conditions showed that, for the YPD and CYM media, the shaking condition had a higher growth rate on the non-woven mats. Notably, the YPD SH media displayed (Figure 3A) an unexpected order among the dilutions, possibly due to the unoptimized sterilization protocol, which led to inconsistent differences in coverage percentages and artificially lowered the overall mean.

**Figure 4:**
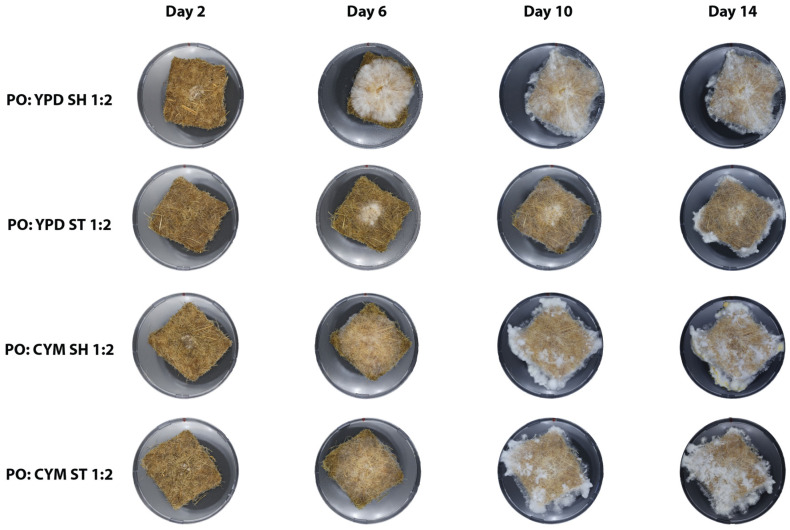
Growth of *P. ostreatus* mycelium on non-woven hemp mats over a 14-day period. The columns represent different time points, while the rows show the effect of different preculture conditions in which the mycelium was initially grown.

From a visual analysis (Figure 4), *P. ostreatus* developed thin hyphae that spread across the mat until they reached the edges. By day 10, it began to produce aerial mycelium, which appeared as a white fluffy substance extending beyond the perimeter of the mats. Notably, *P. ostreatus* did not grow densely, allowing the non-woven structure of the mat to remain visible through the hyphae.

#### 3.2.2. Growth Kinetics of *G. lucidum* on Non-Woven Hemp Mats

**Figure 5:**
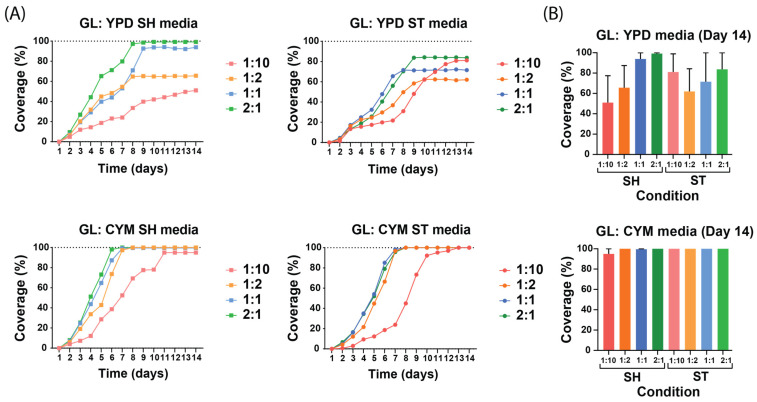
Growth curves of *G. lucidum* under different preculture conditions and dilutions. All values are presented as mean from n ≥ 2 independent samples and 1:10 to 2:1 indicate the dilution of mycelium in the liquid media. (**A**) Growth curves of liquid spawn of each medium and each condition. The growth curves highlight how these variables affect the growth kinetics across different media and conditions. (**B**) Coverage percentage of all static and shaking conditions on the 14th day of growth of *G. lucidum* in YPD and CYM media.

When comparing liquid media conditions for *G. lucidum* (Figure 5), CYM media clearly outperformed YPD. CYM achieved 100% coverage for both conditions and all dilutions. All conditions were able to achieve 100% coverage, except for the YPD ST condition. In CYM media, the *G. lucidum* species exhibited more consistent results in terms of growth rate. Comparing the shaking and static conditions for both media, it showed that shaking conditions achieved their maximum coverage between day 5 and day 8, whereas it was between day 7 and day 9 for ST.

Comparing all four conditions and dilutions, the same relationship as *P. ostreatus* was observed during the exponential phase of the growth curve. Notably, the YPD ST media displayed an unexpected order among the dilutions similar to *P. ostreatus* YPD SH. YPD growth curves displayed distinct separation, with each curve reaching specific growth percentage levels at different rates. The CYM dilutions of 1:2, 1:1, and 2:1 showed similar growth curves, while dilution 1:10 had a noticeably lower growth curve.

**Figure 6:**
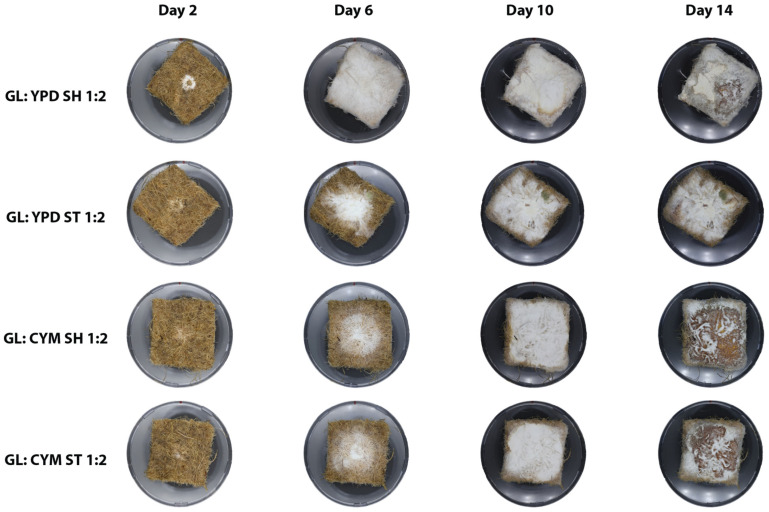
Growth of *G. Lucidum* on non-woven hemp mats over a 14-day period. The columns represent different time points, while the rows show the effect of different preculture conditions in which the mycelium was initially grown.

*G. lucidum* primarily grew within the mat area and did not produce aerial mycelium (Figure 6). It exhibited a denser growth pattern, with the mat’s structure covered by hyphae. Additionally, *G. lucidum* underwent color changes. For both YPD ST and SH conditions, *G. lucidum* started to change color between days 6 and 13, whereas in CYM ST and CYM SH, the color changes began between days 8 and 11.

## 4. Discussion

The understanding of the liquid spawn as a design parameter will open digital biofabrication possibilities to produce MBC. The results of this experiment are a first indication that the preculture media influences biomass growth and that the biomass in a liquid spawn can be used as a parameter to control mycelium growth through dilution. Liquid spawn offers the potential for precise deposition and mixing within the mats.

### 4.1. Organic Substrate Is Susceptible to Contamination

The effectiveness of mycelium growth is dependent on the initial mycelium biomass concentration. Less deposited biomass will promote slower growth of mycelium, making the substrate more prone to contamination [28], though higher concentrations could potentially be achieved with liquid spawn if allowed to grow for a longer period, in a shaking condition or when an optimized nutrient medium is made [29].

**Figure A3:**
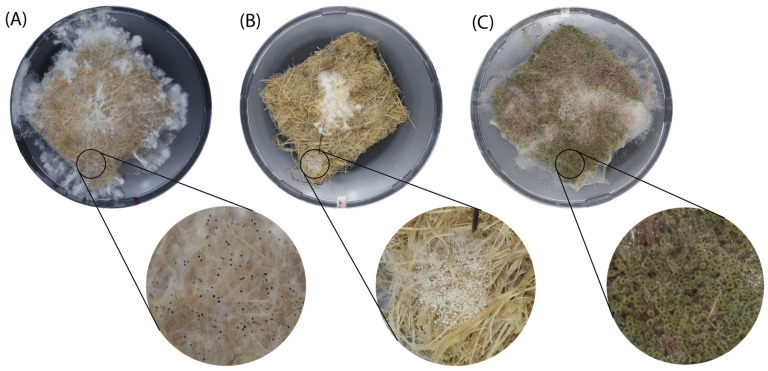
Three occurring contaminations during the experiment. (**A**) Black variant, (**B**) white variant, and (**C**) green variant. No analysis has been conducted on which microorganisms these are.

During our experiments, contaminants were observed in the technical repeats during the incubation of the liquid spawn on the mats, probably arising from unoptimized sterilization processes. The sterilization method was modified to sterilize for longer times, which resulted in less contamination, aligning with previous research [1]. Still, some repeats showed contamination, resulting in decreased or halted mycelium growth. All contaminated samples were excluded from the test results; yet, enough technical repeats were produced to gain insightful results. The observed contamination appeared consistent with the characteristics of the *Aspergillus* genus, a group of molds commonly found in diverse environments worldwide. *Aspergillus* typically exhibits yellow, green, or black coloration, seen in Figure A3 (Appendix A), with a fluffy texture due to spore production, supporting the assumption that this was the contaminant [30]. Further investigation of the sterilization protocol is of the utmost importance to fabricate MBCs without interference.

### 4.2. Precultures Have an Influence on Mycelium Growth

The results from both ST and SH conditions, along with their respective dilutions, provide valuable insights as a foundation for the digital biofabrication system. The shaking condition aligns with previous research, where mycelium tends to grow in pellets due to the centrifugal forces exerted within the liquid media, increasing mycelium biomass [29,31]. This impacts both the system’s energy use and the controllability of mycelium growth. Shaking conditions enhance biomass production through continuous oxygen exchange with the liquid, as demonstrated in our findings with *P. ostreatus* and *G. lucidum*, where shaking led to improved growth rates on the mats [31]. However, shaking cultures must be vortexed before inoculation to prevent clogging caused by spherical growth forms, which can interfere with deposition or injecting the liquid spawn. This extra step increases energy consumption during fabrication, making static growth preferable, as it also supports effective mycelium development without the additional energy input.

The growth curves showed that CYM medium improved growth on hemp mats over YPD. Mycelium growth is dependent on the carbon-to-nitrogen ratio (C/N) within the medium. The results indicated that a lower C/N ratio, due to a higher amount of nitrogenous material, led to increased biomass production [29]. Since CYM has less nitrogenous material, it may have encouraged the mycelium to develop more adaptive efficient growth forms. When inoculated onto the hemp mats, this adaptation could help the mycelium use the substrate’s nutrients more effectively, resulting in more effective colonization. To control the mycelium during fabrication it is preferred to have slow growth to have time for manipulation. Static conditions may offer more promise for practical use, as shaking precultures require additional energy to produce and the accelerated growth on the hemp mat reduces control over the mycelium, which could be a disadvantage in applications requiring precise growth manipulation. YPD media is therefore preferred over CYM and a higher dilution is recommended, resulting in a slower growth rate while still achieving comparable coverage percentages.

An important parameter for optimizing the digital biofabrication process is the nutrient composition in the liquid spawn, as it directly influences the growth rate and surface coverage of the mycelium. By adjusting nutrient dilution, the digital biofabrication system can precisely control whether the application requires rapid or slow growth, along with full or partial surface coverage.

Key findings indicate that higher dilutions allow for slower growth and partial coverage, enabling finer control when precise growth patterns are needed. Lower dilutions, on the other hand, support rapid growth and complete surface coverage. Interestingly, the study shows that higher dilutions can sometimes achieve similar coverage percentages to lower ones, making it possible to generate more preculture from a single batch. This discovery has significant implications for material efficiency and cost reduction in production, enhancing the flexibility and sustainability of the digital biofabrication system. By integrating nutrient composition adjustments as a variable, the digital biofabrication process becomes a more versatile and cost-effective tool for creating customized MBCs.

## 5. Conclusions

This study proves feasibility and showcases potential for using liquid spawn for digital biofabrication. It provides an initial framework for growing and applying liquid spawn on non-woven hemp mats, with findings that underscore the potential to produce mycelium based composites (MBCs) for building interior applications. By examining liquid spawn, we demonstrate that liquid spawn offers precision in deposition and growth control on non-woven substrates, making it well suited for digital biofabrication systems. Our results show that liquid spawn can produce both fast and slow growth, along with localized and uniform growth. Precultures grown under shaking conditions enhance growth on non-woven mats but could increase overall energy use and fabrication costs. Lower mycelium dilutions in liquid spawn show faster growth rates and higher coverage, with CYM media more effectively promoting mycelium growth than YPD. Additionally, a higher dilution allows better control over growth due to its slower growth rate, a factor beneficial for digital biofabrication as it enables fine-tuning of material properties. The adaptability of mycelium growth is observed, particularly with lower dilutions and CYM media, which promote rapid growth and improve coverage on the hemp mats.

Furthermore, liquid spawn’s compatibility with different growth kinetics potentially supports targeted growth manipulation, enabling a promising route for creating property-specific MBCs. Future research will aim to develop optimized protocols for applying liquid spawn with digital biofabrication, investigate multi-species composites to enhance specific material properties, and refine digital biofabrication systems for precise and localized growth control, enabling the production of materials directly through these advanced biofabrication systems. The next step will involve characterizing the material properties of MBCs fabricated with liquid inoculation. Together, these efforts aim to broaden MBC applications in interior architecture, enhancing the material’s functionality, sustainability, and design potential.

## Data Availability

Data supporting the findings of this study are available upon request.
